# Illuminating radiogenomic signatures in pediatric-type diffuse gliomas: insights into molecular, clinical, and imaging correlations. Part II: low-grade group

**DOI:** 10.1007/s11547-025-02049-0

**Published:** 2025-07-16

**Authors:** Ryo Kurokawa, Akifumi Hagiwara, Rintaro Ito, Daiju Ueda, Tsukasa Saida, Akihiko Sakata, Kentaro Nishioka, Shunsuke Sugawara, Koji Takumi, Tadashi Watabe, Satoru Ide, Mariko Kawamura, Keitaro Sofue, Kenji Hirata, Maya Honda, Masahiro Yanagawa, Seitaro Oda, Mami Iima, Shinji Naganawa

**Affiliations:** 1 Department of Radiology, Graduate School of Medicine, The University of Tokyo, 7-3-1 Hongo, Bunkyo-Ku, Tokyo, 113-8655 Japan; 2 Department of Radiology, Juntendo University School of Medicine, Tokyo, Japan; 3 Department of Radiology, Nagoya University Graduate School of Medicine, Nagoya, Japan; 4 Department of Artificial Intelligence, Graduate School of Medicine, Osaka Metropolitan University, Osaka, Japan; 5 Department of Radiology, Institute of Medicine, University of Tsukuba, Tsukuba, Japan; 6 Department of Diagnostic Imaging and Nuclear Medicine, Kyoto University Graduate School of Medicine, Kyoto, Japan; 7 Department of Radiation Oncology, Graduate School of Medicine, Hokkaido University, Sapporo, Japan; 8 Department of Diagnostic Radiology, National Cancer Center Hospital, Tokyo, Japan; 9 Department of Radiology, Graduate School of Medical and Dental Sciences, Kagoshima University, Kagoshima, Japan; 10 Department of Radiology, Osaka University Graduate School of Medicine, Osaka, Japan; 11 Department of Radiology, School of Medicine, University of Occupational and Environmental Health, Kitakyushu, Japan; 12 Department of Radiology, Kobe University Graduate School of Medicine, Hyogo, Japan; 13 Department of Diagnostic Imaging, Graduate School of Medicine, Hokkaido University, Sapporo, Japan; 14 Department of Diagnostic Radiology, Kansai Electric Power Hospital, Osaka, Japan; 15 Department of Diagnostic Radiology, Graduate School of Medical Sciences, Kumamoto University, Kumamoto, Japan

**Keywords:** Pediatric-type, diffuse glioma, MYB, MAPK, PLNTY, radiology-molecular correlation

## Abstract

The fifth edition of the World Health Organization classification of central nervous system tumors represents a significant advancement in the molecular-genetic classification of pediatric-type diffuse gliomas. This article comprehensively summarizes the clinical, molecular, and radiological imaging features in pediatric-type low-grade gliomas (pLGGs), including MYB- or MYBL1-altered tumors, polymorphous low-grade neuroepithelial tumor of the young (PLNTY), and diffuse low-grade glioma, MAPK pathway-altered. Most pLGGs harbor alterations in the RAS/MAPK pathway, functioning as “one pathway disease”. Specific magnetic resonance imaging features, such as the T2-fluid-attenuated inversion recovery (FLAIR) mismatch sign in MYB- or MYBL1-altered tumors and the transmantle-like sign in PLNTYs, may serve as non-invasive biomarkers for underlying molecular alterations. Recent advances in radiogenomics have enabled the differentiation of BRAF fusion from BRAF V600E mutant tumors based on magnetic resonance imaging characteristics. Machine learning approaches have further enhanced our ability to predict molecular subtypes from imaging features. These radiology-molecular correlations offer potential clinical utility in treatment planning and prognostication, especially as targeted therapies against the MAPK pathway emerge. Continued research is needed to refine our understanding of genotype–phenotype correlations in less common molecular alterations and to validate these imaging biomarkers in larger cohorts.

## Introduction

The fifth edition of the World Health Organization Classification of Tumors of the Central Nervous System (WHO CNS5), published in 2021, represents a noteworthy advancement in the molecular-genetic classification framework for CNS neoplasms [1]. This revised taxonomy incorporates several substantial modifications, including the implementation of deoxyribonucleic acid (DNA) methylation-based diagnostic methodologies, the stratification of diffuse gliomas into distinct pediatric and adult molecular subtypes, and the recognition of twenty-two novel tumor entities. These refinements reflect the integration of contemporary molecular insights into neuro-oncological classification systems, thereby enhancing diagnostic precision and potentially facilitating more targeted therapeutic approaches for patients with central nervous system malignancies [2–4]. The classification of diffuse gliomas into pediatric-type and adult-type in WHO CNS5 represents a landmark change in understanding CNS tumors. Pediatric-type diffuse gliomas are now categorized into four high-grade types (diffuse midline glioma (DMG), H3 K27-altered; diffuse hemispheric glioma, H3 G34-mutant; diffuse pediatric-type high-grade glioma, H3-wildtype and IDH-wildtype; and infant-type hemispheric glioma) and four low-grade types (Tables 1 and 2; diffuse astrocytoma, MYB- or MYBL1-altered; angiocentric glioma (AG); polymorphous low-grade neuroepithelial tumor of the young (PLNTY); and diffuse low-grade glioma, MAPK pathway-altered (DLGG-MAPK)).

**Table 1:** Abbreviations of the tumors

AG	Angiocentric glioma
DLGG-MAPK	Diffuse low-grade glioma, MAPK pathway-altered
DMG	Diffuse midline glioma
DNET	Dysembryoplastic neuroepithelial tumor
LEAT	Long-term epilepsy-associated tumor
pHGG	Pediatric-type high-grade glioma
pLGG	Pediatric-type low-grade glioma
PLNTY	Polymorphous low-grade neuroepithelial tumor of the young

**Table 2:** Radiological characteristics of pediatric-type low-grade gliomas

Tumor type	CNS WHO grade	Molecular characteristics	Common age/location	General radiological findings
Diffuse astrocytoma, MYB- or MYBL1-altered	1	MYB or MYBL1 rearrangements	- Primarily in children (median age: 5–7 years) - Usually located in supratentorial region	- T2-weighted hyperintense, well-defined - May show T2-FLAIR mismatch sign - Minimal contrast enhancement
Angiocentric glioma	1	MYB rearrangements (most often: MYB::QKI fusion)	- Supratentorial (cortical and subcortical areas) (median age: 13 years) - Some brainstem cases (median age: 5 years)	- Cortical and subcortical location - Intratumoral T1-weighted hyperintensity - Stalk-like sign - May show T2-FLAIR mismatch sign - May lack contrast enhancement
Polymorphous low-grade neuroepithelial tumor of the young	1	BRAF V600E mutation FGFR2/FGFR3 rearrangements	- Mid-to-late teens, but wide range (4–60 + years) - Supratentorial, predominantly in the temporal lobe	- Solid and cystic, well-defined - Frequent calcifications - Transmantle-like sign - No T2-FLAIR mismatch sign - Slight or no contrast enhancement
Diffuse low-grade glioma, MAPK pathway-altered	Not assigned	MAPK pathway alterations	- Primarily in children (< 19 years) - Supratentorial (cortical and subcortical areas), predominantly in the temporal lobe - Most of tectal gliomas belong to this type - Can occur anywhere in the central nervous system	- T2-weighted hyperintense, variable borders - May show T2-FLAIR mismatch sign - Cystic components are frequently observed - Variable contrast enhancement pattern

CNS = central nervous system; WHO = World Health Organization; FLAIR = fluid-attenuated inversion recovery

**Fig. 1:**
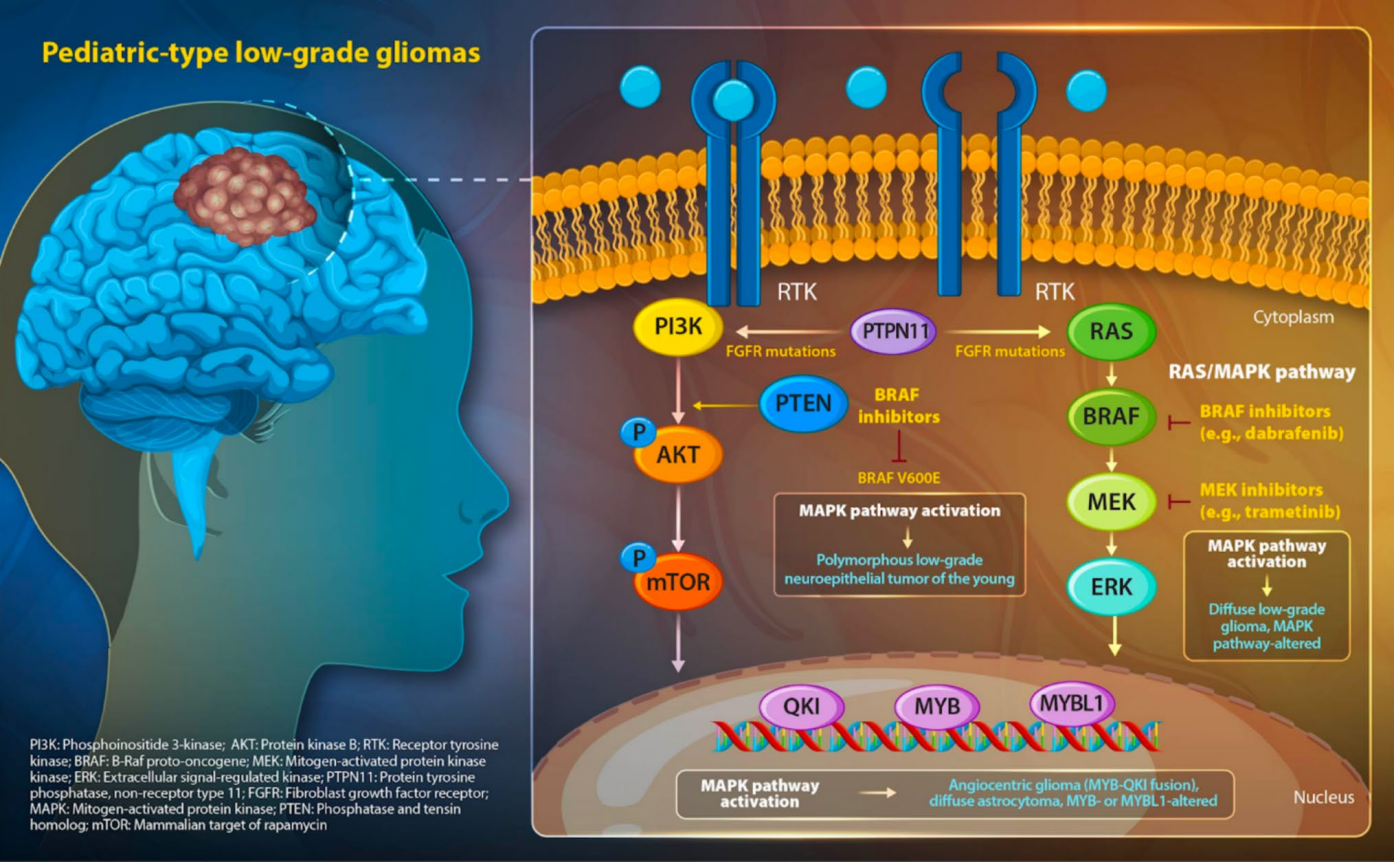
Molecular pathways and targeted therapeutics in pediatric-type low-grade gliomas. Schematic representation of key signaling pathways implicated in pediatric-type low-grade gliomas. Left: Supratentorial low-grade glioma in the pediatric brain. Right: Molecular signaling cascades driving gliomagenesis. Two primary pathways are highlighted: the PI3K/AKT/mTOR pathway (left) and the RAS/MAPK pathway (right). Both pathways can be activated through FGFR mutations or PTPN11. BRAF V600E mutations activate the MAPK pathway, leading to polymorphous low-grade neuroepithelial tumors. Constitutive activation of the MAPK pathway results in diffuse low-grade gliomas, MAPK pathway-altered. Specific genetic alterations produce distinct histological subtypes: MYB or MYBL1 alterations lead to diffuse astrocytomas, MYB- or MYBL1-altered, while MYB-QKI fusion is associated with angiocentric gliomas. Potential therapeutic targets are indicated, including BRAF inhibitors and MEK inhibitors, which block key nodes in these oncogenic signaling cascades

Advances in molecular genetics have revealed that the majority of pediatric-type low-grade gliomas (pLGGs) harbor genetic alterations related to the RAS/MAPK signaling pathway [5]. These include KIAA1549-BRAF fusion genes (most frequent), BRAF V600E mutations, NF1 gene inactivation, other receptor tyrosine kinase (RTK) mutations (FGFR1/2/3, MET, PDGFRA, ALK, ROS1, NTRK2, etc.), and a small number of non-RAS/MAPK pathway alterations (such as MYB or MYBL1 rearrangements). Even in pLGGs with non-RAS/MAPK pathway mutations, elevated levels of phosphorylated ERK, the final product of the RAS/MAPK pathway, have been observed, suggesting indirect activation of the RAS/MAPK pathway [5]. Thus, direct or indirect activation of the RAS/MAPK pathway represents a universal alteration in pLGGs [6]. This concomitantly indicates the potential for targeted therapy directed at the RAS/MAPK pathway, with numerous novel therapeutic approaches under investigation, primarily centered on Food and Drug Administration-approved RAF inhibitors (targeting mutant BRAF) and MEK inhibitors (downstream pathway blockers) (Fig. 1) [7].

MRI remains the gold standard imaging modality for pediatric brain tumors. In parallel with the growing application of artificial intelligence technologies such as radiomics and machine learning in the diagnosis of various tumors, their usefulness in the detection and molecular subtyping of pediatric brain tumors is beginning to gain attention [8–13]. Although the utility of positron emission tomography (PET) in the management of pediatric-type high-grade diffuse gliomas and adult-type diffuse gliomas has become increasingly recognized, its application in pLGGs remains considerably more limited due to the low tracer uptake in pLGGs, which often results in insufficient contrast between tumor and background on PET images [14–19]. Consequently, this relative limitation of PET in pLGGs has further emphasized the importance of MRI in their clinical management.

In this comprehensive review, we elucidate the molecular, clinical, and radiological characteristics of pLGGs, with particular emphasis on the correlative analysis between imaging phenotypes observed on MRI and their corresponding molecular underpinnings.

## Diffuse astrocytoma, MYB- or MYBL1-altered

### General

Diffuse astrocytoma, MYB- or MYBL1-altered is an infiltrative neoplasm of astroglial origin, characterized by uniform cellular morphology and specific genetic modifications in either the MYB or MYBL1 genes. This tumor type was first included in the WHO CNS5 classification. By definition, these tumors do not carry IDH1/IDH2 mutations or H3 gene mutations [1]. These tumors exhibit alterations related to MYB or MYBL1 outside the canonical RAS/MAPK cascade; however, these alterations lead to indirect activation of the RAS/MAPK pathway [5, 20]. Recent research has revealed that both gene fusions and truncations without productive fusion can result in the development of this tumor type [21]. Diffuse astrocytoma, MYB- or MYBL1-altered, is classified as CNS WHO grade 1 with favorable prognosis, with a reported 5-year event-free survival rate of 100% in cases with near-total resection [20]. A minority of cases may demonstrate progression or metastasis, and in these aggressive variants, there is evidence of activation of both the MAPK and PI3K/AKT/mTOR pathways [20].

Both diffuse astrocytoma, MYB- or MYBL1-altered and angiocentric glioma (AG) are pLGGs that harbor MYB or MYBL1 alterations. Due to their histological similarities and shared molecular characteristics, there is a growing tendency to consider these entities as part of the same tumor spectrum under the collective designation of "MYB- or MYBL1-altered pLGGs [20]." While these two types of MYB- or MYBL1-altered pLGGs are more common in children (median age 5–13 years), there has been an increasing number of reports in patients over 18 years of age [20, 22]. Many cases present with seizures during childhood (48–81%), placing this tumor within the broad category of long-term epilepsy-associated tumors (LEATs) [20, 23].

The primary therapeutic approach for diffuse astrocytoma, MYB- or MYBL1-altered, is maximal safe surgical resection. However, due to its infiltrative nature, total resection is often not achievable; these patients require careful post-operative monitoring for tumor progression [1]. When complete removal is accomplished, particularly for cortical tumors causing seizures, it frequently results in long-term disease control. In cases where complete resection is not possible or when tumor progression is observed, standard practice dictates initiation of adjuvant therapy. For young children, chemotherapy is preferred as first-line adjuvant treatment to delay or avoid radiation therapy and its associated long-term side effects [24–26]. Common chemotherapeutic regimens include carboplatin plus vincristine, weekly vinblastine, or multi-agent combinations such as TPCV (thioguanine, procarbazine, lomustine, vincristine) therapy [27].

Unlike other pediatric low-grade gliomas (pLGGs), diffuse astrocytoma, MYB- or MYBL1-altered, typically lacks common therapeutic targets such as BRAF mutations. Despite significant advances in the development of BRAF/MEK inhibitors for MAPK-driven pLGGs, specific anti-MYB therapies have not yet been established. Current research is focused on exploring potential targetable pathways downstream of MYB. For the present, conventional therapy remains effective for managing these tumors, with consideration of targeted clinical trials upon disease recurrence.

### Imaging features

**Fig. 2:**
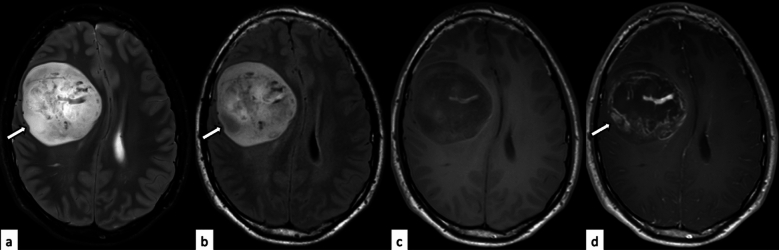
Diffuse astrocytoma, MYB-altered in an 18-year-old man. The tumor mass with well-circumscribed margin in the right frontal lobe shows hyperintensity on fat-saturated T2-weighted imaging (**a**) and fluid-attenuated inversion recovery (FLAIR) imaging (**b**), heterogeneous hypointensity on T1-weighted imaging (**c**), and heterogeneous contrast enhancement on contrast-enhanced T1-weighted imaging (**d**). Partial T2-FLAIR mismatch sign (**a**, **b**, arrows) is observed within the contrast-enhanced component (**d**, arrow), indicating that it is not a cystic degeneration. The same case with different images has been evaluated in our previous study [2]

In most cases of diffuse astrocytoma, MYB- or MYBL1-altered form masses in the supratentorial region, although brainstem involvement occurs only exceptionally [1, 20]. Key imaging characteristics include well-defined borders, low attenuation on non-contrast computed tomography (CT), hyperintensity on T2-weighted imaging (T2WI) with or without T2-fluid-attenuated inversion recovery (FLAIR) mismatch sign, absence of diffusion restriction, and minimal to no contrast enhancement (Fig. 2) [2, 28]. In cases originating in the brainstem, differentiation from diffuse midline glioma (DMG), H3K27-altered, may be challenging without information regarding growth rate [33]. It should be noted that while the T2-FLAIR mismatch sign was originally validated in non-enhancing tumors, subsequent literature has shown varying approaches to its application, with some studies including tumors with contrast enhancement [29–32]. The evolving criteria and ongoing debate regarding strict versus relaxed application of this imaging feature should be considered when interpreting these findings. In this article, the T2-FLAIR mismatch sign is defined as positive when lesions exhibit homogeneous hyperintense signal on T2-weighted imaging with hypointense signal on FLAIR imaging except for a hyperintense thin peripheral rim, regardless of the presence of contrast enhancement.

### Radiology-molecular correlation

MYB or MYBL1 alterations represent driver mutations that occur early in the tumorigenesis process of diffuse astrocytoma, MYB or MYBL1-altered, resulting in the presence of this abnormality in nearly all tumor cells. Molecular changes that are ubiquitously present throughout the tumor cell population can be expected to manifest as discernible features on imaging studies.

In the pediatric population, the T2-FLAIR mismatch sign has been suggested as a potential imaging biomarker for MYB- or MYBL1-altered tumors. This radiological finding refers to lesions that display nearly homogeneous hyperintensity on T2WI, while appearing hypointense throughout most of the lesion except at the periphery on FLAIR images (excluding areas of cystic degeneration or necrosis). Histologically, this sign is believed to reflect microcystic changes within the tumor and has traditionally been recognized as a highly specific imaging finding for IDH-mutant astrocytomas in adult-type diffuse gliomas [34].

Recent studies have demonstrated that the T2-FLAIR mismatch sign can also be observed in brain tumors other than IDH-mutant astrocytomas, including pediatric high-grade diffuse gliomas such as diffuse midline glioma, H3K27-altered, and various pLGGs [35, 36]. van Maren et al. [28] reported that the T2-FLAIR mismatch sign was more frequently observed in MYB- or MYBL1-altered tumors compared to IDH-mutant astrocytomas in the pediatric population.

## Angiocentric glioma

### General

AG is histologically characterized by thin, cytologically bland, bipolar cells that aggregate at least partially in perivascular spaces. The molecular pathogenesis of AG has been elucidated, with most tumors harboring the characteristic MYB::QKI fusion. This genetic alteration is thought to contribute to tumorigenesis through three distinct mechanisms [37]: (1) MYB activation by truncation, (2) enhancer translocation driving aberrant MYB-QKI expression, and (3) hemizygous loss of the tumor suppressor QKI.

In clinical practice, the diagnostic approach for suspected AG begins when imaging suggests a low-grade glioma in a pediatric patient or young adult. Subsequently, a biopsy or resection is performed to obtain tissue for histopathological examination. The diagnosis of AG is confirmed by identifying its characteristic perivascular growth pattern and testing for MYB arrangements. The detection of an MYB alteration, particularly the MYB::QKI fusion, serves as a critical molecular diagnostic marker, as this rearrangement is highly specific for angiocentric glioma [1]. However, recent histopathological observations have revealed greater complexity in AG morphology. A study reported that all AGs exhibited areas resembling diffuse astrocytoma or isomorphic diffuse astrocytoma at the infiltrating edges, where tumor cell densities were lower [20]. This histological overlap between AG and diffuse astrocytoma, MYB- or MYBL1-altered pose challenge in differential diagnosis. As mentioned previously, there is a trend toward collectively managing AG and diffuse astrocytoma, MYB- or MYBL1-alterations within the same spectrum, under the broader classification of MYB- or MYBL1-altered pLGGs [20].

AG is classified as CNS WHO grade 1 with favorable prognosis. Gross total resection of AG is usually curative and significantly improves seizure outcomes, with adjuvant therapy rarely being necessary [38]. When complete removal is achieved, it typically results in long-term disease control, especially for cortical tumors causing seizures.

### Imaging features

AG predominantly demonstrates cortico-subcortical localization in the supratentorial region; however, approximately 14% of cases primarily involve the brainstem [22]. An important clinical distinction exists between brainstem and supratentorial AGs: patients with brainstem AG tend to be significantly younger than those with supratentorial AG (median age: 5 years [range: 2–7 years] vs. 13 years [range: 2–83 years]) [22]. While supratentorial AGs typically present with seizures, brainstem AGs tend to manifest with cranial nerve palsies or signs of increased intracranial pressure [22].

**Fig. 3:**
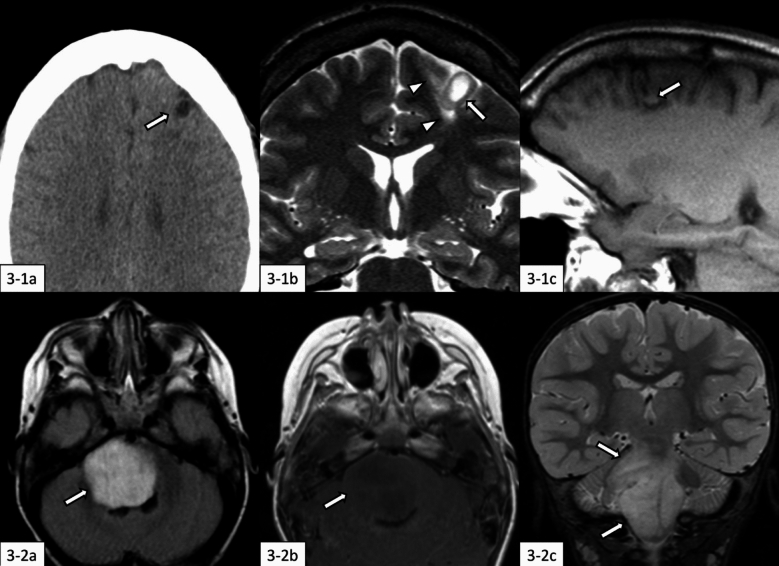
Angiocentric glioma. **3-1** (43-year-old man): There is a tumor mass with the cortical and subcortical involvement in the right frontal lobe (**3-1a**–**c**, arrows). The tumor mass shows hypodensity on non-contrast computed tomography (**3-1a**), hyperintensity on T2-weighted coronal imaging (**3-1b**), hyperintense rim and central hypointensity on T1-weighted sagittal imaging (**3-1c**). The hyperintense “stalk-like sign” in the surrounding brain parenchyma toward the lateral ventricle is observed on T2-weighted coronal image (**3-1b**, arrowheads). **3-2** (2-year-old boy): There is a tumor mass in the pontomedullary region (**3-2a**–**c**, arrows). The tumor mass shows hyperintensity on fluid-attenuated inversion recovery (**3-2a**) and hypointensity without contrast enhancement on contrast-enhanced T1-weighted imaging (**3-2b**). Fat-saturated T2-weighted coronal imaging shows infiltrating growth of the tumor mass with an ill-defined margin (**3-2c**), mimicking diffuse midline glioma, H3K27-altered. The same cases with different images have been evaluated in our previous study [22]

AG exhibits several distinctive radiological features: approximately 46% of cases show intratumoral high-intensity areas on T1-weighted imaging (T1WI), 20% demonstrate T2WI/FLAIR high-intensity lesions extending to the ventricle (known as the “stalk-like sign”), and 26.8% of cases display predominantly heterogeneous enhancement (Fig. 3) [22]. Brainstem AG, similar to brainstem diffuse astrocytoma, MYB- or MYBL1-altered, can mimic DMG, H3K27-altered [22].

The absence of seizures as a presenting symptom in brainstem AG is attributed to the lack of cerebral cortical involvement, which is typically required to trigger seizure activity. Several hypotheses have been proposed to explain the earlier diagnosis of brainstem AGs compared to their supratentorial counterparts: supratentorial AGs may be overlooked on initial imaging; brainstem AGs present with symptoms such as vomiting or cranial nerve deficits that more readily prompt radiological examination, resulting in earlier diagnosis; or there may exist undiscovered molecular differences that account for variations in tumor location and age of onset.

The unique radiological features of AG, including intratumoral T1 hyperintensity and the “stalk-like sign” on T2WI/FLAIR, may reflect the distinctive histological architecture of these tumors, particularly their angiocentric growth pattern and the associated perivascular orientation of tumor cells. Further research is needed to elucidate the precise molecular mechanisms underlying these radiological characteristics and to determine their potential utility as non-invasive biomarkers for tumor diagnosis and monitoring.

### Radiology-molecular correlation

The radiology-molecular correlation of AG is essentially the same as that of the aforementioned diffuse astrocytoma, MYB- or MYBL1-altered.

## Polymorphous low-grade neuroepithelial tumor of the young (PLNTY)

### General

PLNTY is a CNS WHO grade 1 brain tumor characterized by diffuse growth patterns, frequent presence of oligodendroglioma-like components, calcification, CD34 immunoreactivity, and MAPK pathway-activating genetic abnormalities. It was first included in the WHO CNS5 classification [1]. Although PLNTY histologically resembles oligodendroglioma, it differs from oligodendroglioma, IDH-mutant and 1p/19q-codeleted in that it is IDH-wildtype and lacks 1p/19q-codeletion.

The median age at diagnosis is mid to late teens, but cases have been reported across a wide age range from 4 to over 60 years. Most patients experience refractory epileptic seizures ranging from one month to up to 18 years prior to diagnosis. For this reason, this tumor is also classified within the category of LEATs. Complete surgical resection typically results in favorable outcomes and often improves seizure control.

PLNTYs generally harbor mutually exclusive BRAF V600E mutations or FGFR2/FGFR3 rearrangements, both related to the MAPK pathway [39]. BRAF V600E-positive PLNTYs, compared to negative cases, tend to occur more frequently in patients over 18 years of age (21/31 [67.7%] vs. 7/31 [22.6%]) and involve the temporal lobe (27/31 [87.1%] vs. 17/31 [54.8%]) [40].

While PLNTYs generally follow a benign course, PLNTYs with FGFR3-TACC3 fusion are known to exhibit histologic features similar to high-grade gliomas and malignant molecular features (CDKN2A loss, TERT promoter mutations, and lack of MGMT promoter methylation), with reports of malignant transformation [41, 42].

The cornerstone of treatment is maximal safe surgical resection. Gross total resection is the goal and is often curative, while improving seizure control. One series reported up to 90% of patients became seizure-free after surgery [43]. Given that nearly all cases harbor a driver mutation—typically a BRAF alteration (most often the activating BRAF V600E mutation) or alternative MAPK pathway fusions involving FGFR2, FGFR3, or NTRK2, targeted therapies with RAF inhibitors and MEK inhibitors could be promising [7]. Preclinical models showed that combining BRAF inhibitors with MEK inhibitors achieves more durable pathway blockade, translating into prolonged tumor control and survival benefits in vivo [44]. For PLNTY patients with BRAF fusions or uncommon BRAF mutations (where BRAF inhibitor dabrafenib, which targets V600E, would not apply), pan-RAF Inhibitor tovorafenib offers a more tailored targeted approach [45].

### Imaging features

**Fig. 4:**
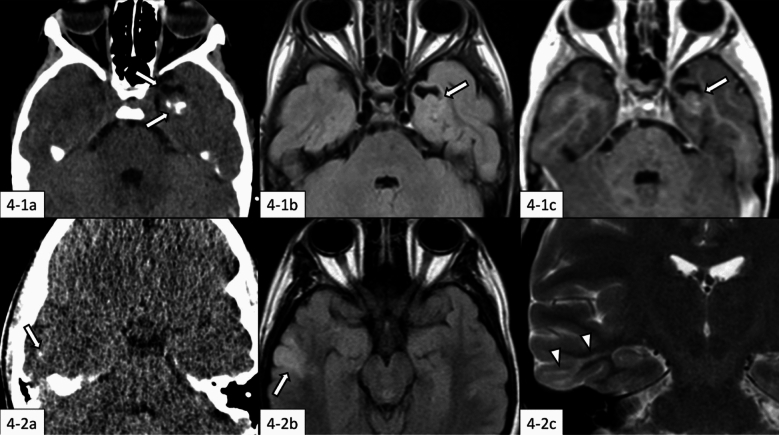
Polymorphous low-grade neuroepithelial tumor of the young. **4-1** (7-year-old girl): There is a solid and cystic tumor mass involving the cortical and subcortical regions in the left temporal lobe (**4-1a**–**c**, arrows). The tumor mass shows solid calcification on non-contrast computed tomography (CT) (**4-1a**), isointensity to the cortex on fluid-attenuated inversion recovery (FLAIR) imaging (**4-1b**), and scarce enhancement on contrast-enhanced T1-weighted imaging (**4-1c**). The same case with different images has been evaluated in our previous study [2]. **4-2** (17-year-old girl): There is a tumor mass involving the cortical and subcortical regions in the right temporal lobe (**4-2a**, **4-2b**, arrows). The tumor mass shows punctate calcification on non-contrast CT (**4-2a**), hyperintensity on FLAIR imaging (**4-2b**) with hyperintense “transmantle-like sign” extending from the cortex to the ventricle on T2-weighted coronal imaging (**4-2c**, arrowheads). The same case with different images has been evaluated in our previous study [47]

PLNTYs are typically located supratentorially, with the temporal lobe being the most frequent site. Almost all tumors extend into the cortex or subcortical white matter. These tumors usually have well-defined borders, frequently contain calcifications, and often present with both solid and cystic components (Fig. 4). The calcifications typically appear punctate in childhood cases and tend to be coarser in adults, suggesting the possibility of progression over time [46].

Approximately half of the cases demonstrate T2WI/FLAIR hyperintensity that is either confined to the peritumoral region or extends toward the ventricle ("transmantle-like sign") [46, 47].

### Radiology-molecular correlation

The transmantle-like sign refers to T2WI/FLAIR hyperintensity that extends from the peritumoral region toward the ventricle, representing a characteristic MRI finding observed in approximately half of PLNTYs. Limited pathological correlation studies have demonstrated that this sign corresponds to tumor infiltration and gliosis, with or without cortical dysplasia [47]. The specific name “transmantle-like sign” derives from its similarity to the “transmantle sign”, which was originally reported as a characteristic MRI finding in cortical dysplasia [48]. Similar imaging findings have been reported in other LEATs, such as the “stalk-like sign” in AG, and comparable features without specific nomenclature have been noted in ganglioglioma and dysembryoplastic neuroepithelial tumor (DNET) [22].

LEATs share the common clinical presentation and localization despite histological and molecular genetic differences: early-onset, predilection for the temporal lobe, benign course classified as CNS WHO grade 1, slow growth tendency, and long-term seizure history (typically > 2 years). Interestingly, LEATs other than PLNTY, including ganglioglioma, DNET, and MYB- or MYBL1-altered pLGGs, also frequently coexist with regional cortical dysplasia [49, 50]. Furthermore, CD34, an intercellular adhesion protein transiently expressed in early neural development, which is diffusely expressed in PLNTY, is known to be frequently positive in cortical dysplasia, especially in type IIb that tends to show transmantle signs, and also partially positive in other LEATs besides MYB- or MYBL1-altered pLGGs [39, 51].

In summary, although the precise mechanism remains unclear, the transmantle(-like) sign or stalk-like sign may be considered a common imaging finding in LEATs, a group of tumors that exhibit similar clinical and radiological characteristics.

## Diffuse low-grade glioma, MAPK pathway-altered

### General

Diffuse low-grade glioma, MAPK pathway-altered (DLGG-MAPK), is an IDH-wildtype, H3-wildtype, low-grade glioma with diffuse astrocytic or oligodendroglial morphology characterized by a pathogenic alteration in a gene that codes for a MAPK pathway protein. This tumor type was first included in the WHO CNS5 and has not been assigned a specific CNS WHO grade [1].

This tumor typically occurs in pediatric patients and can arise anywhere in the CNS, including the cerebral hemispheres, diencephalon, brainstem, cerebellum, and spinal cord. Missense mutations, intragenic duplications, or fusions may constitutively activate a receptor tyrosine kinase, such as FGFR or NTRK, leading to aberrant recruitment of the G protein RAS, which activates the RAF/MEK/ERK cascade (MAPK pathway), resulting in tumorigenesis [52, 53]. Among the MAPK pathway alterations in DLGG-MAPK, those resulting from BRAF p.V600E mutation, KIAA1549-BRAF fusion, or FGFR1 alteration occur with the highest frequency [5].

The initial standard therapy is maximal safe surgical resection whenever feasible, since surgery alone can often control circumscribed tumors. When residual tumor remains, front-line adjuvant therapy is typically chemotherapy rather than radiation, as with other pediatric-type diffuse gliomas. Numerous targeted therapies for DLGG-MAPK are being explored to improve outcomes and reduce long-term toxicity. The two main classes are RAF inhibitors (targeting mutant BRAF) and MEK inhibitors (downstream pathway blockers). Beyond RAF/MEK, precision therapies targeting less common alterations are under investigation. These include FGFR1/FGFR2 inhibitors for tumors with FGFR fusions or mutations, TRK inhibitors for NTRK fusion-positive gliomas, and ALK/ROS1 inhibitors for rare fusion-driven cases [54]. Immunotherapy strategies (immune checkpoint blockades, vaccines, chimeric antigen receptor T-cells, or oncolytic viruses) are largely uncharted in DLGG-MAPK, though case reports and small studies are beginning to appear.

### Imaging features

**Fig. 5:**
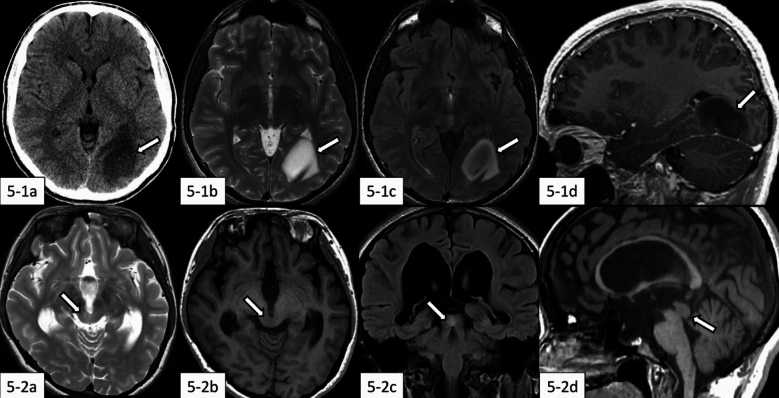
Diffuse low-grade glioma, MAPK pathway-altered. **5-1** (15-year-old boy): There is a tumor mass in the left occipital lobe (**5-1a**–**d**, arrows). The tumor mass shows hypointensity on non-contrast computed tomography (**5-1a**), hyperintensity on T2-weighted imaging (**5-1b**), peripheral hyperintensity on fluid-attenuated inversion recovery (FLAIR) imaging (**5-1c**) with positive T2-FLAIR mismatch sign, and lack of contrast enhancement on contrast-enhanced T1-weighted sagittal imaging (**5-1d**). The same case with different images has been evaluated in our previous study [2]. **5-2** (20-year-old woman): There is a tumor mass in the tectum of the midbrain, accompanied by hydrocephalus (**5-2a**–**d**, arrows). The tumor mass shows hyperintensity on T2-weighted imaging (**5-2a**) and FLAIR coronal imaging (**5-2c**), and hypointensity on T1-weighted axial and sagittal imaging (**5-2b** and **5-2d**, respectively) with ill-defined margin

Reports on the imaging characteristics of DLGG-MAPK are limited. According to a few available studies, these tumors present as T2WI hyperintense masses with either well-defined or ill-defined borders, and they may demonstrate the T2-FLAIR mismatch sign, with or without contrast enhancement (Fig. 5) [2, 55]. Cystic components are frequently observed [56].​

Notably, many tumors previously classified as "tectal gliomas" appear to belong to this type [57, 58]. Chiang et al. [58] investigated genetic alterations in tectal gliomas and reported a high frequency of hotspot KRAS G12R mutations (19/23, 82.6%) and BRAF alterations (14/23, 60.9%), while none had alterations in IDH1/2, TP53, ATRX, histone H3 genes, MYB, or MYBL1. The same study further reported that "methylome profiles of tectal gliomas form a distinct cluster on the t-SNE plot regardless of the mutation status (KRAS only, BRAF only, or combined KRAS/BRAF alterations) [58]." This suggests that tectal gliomas, which morphologically resemble pilocytic astrocytoma, exhibit a methylome profile different from pilocytic astrocytoma. Many of tectal gliomas are now considered to be DLGG-MAPK, a newly included tumor type in the WHO CNS5 [58].

### Radiology-molecular correlation

pLGGs are generally recognized as a "one-pathway disease," with a single, direct or indirect MAPK pathway alteration serving as the main driver of tumorigenesis in most cases [5]. Similar to MYB- or MYBL1-altered pLGGs, DLGG-MAPK tumors are expected to display consistent imaging characteristics throughout the tumor, potentially allowing radiologists to identify characteristic features macroscopically on MRI.

Trasolini et al. [59] compared MRI features of 30 BRAF fusion, 19 BRAF V600E mutant, and 21 wild-type pLGGs, finding that BRAF fusion tumors were significantly larger, had a greater mass effect, more frequently caused hydrocephalus, and showed diffuse enhancement compared to BRAF V600E-mutant tumors. BRAF V600E-mutant tumors were more often hemispheric, appeared more infiltrative, and were the only group demonstrating diffusion restriction, although infrequently. Similarly, Shrot et al. [52] reported that BRAF fusion pLGGs exhibited well-defined tumor margins compared to BRAF V600E-mutant pLGGs.

In a radiogenomics study classifying pLGGs into three groups (BRAF fusion, BRAF mutation, non-BRAF alteration) using FLAIR images, Kudus et al. [60] utilized MRI data from 336 patients. They extracted a large amount of quantitative features from the images (radiomic features) and used them as inputs for machine learning models (radiomics). They also utilized convolutional neural networks, which are deep learning models that automatically learn hierarchical features from image data. Furthermore, they compared a hybrid model that combined both approaches. The hybrid model demonstrated the highest performance (area under the receiver operating characteristic curve (AUC): 0.824), followed by radiomics alone (AUC: 0.802), with the convolutional neural network showing the lowest performance (AUC: 0.764).

Radiology-molecular correlations related to other major driver mutations in DLGG-MAPK, such as KRAS mutations or FGFR alterations, remain insufficiently developed, and further knowledge accumulation is anticipated in the future.

## Conclusion

The integration of molecular genetic insights with radiological features has revolutionized our understanding and classification of pLGGs. The WHO CNS5, with its emphasis on molecular signatures, provides unprecedented precision in diagnosing these tumor types. Characteristic imaging features, such as the T2-FLAIR mismatch sign in MYB- or MYBL1-altered tumors, transmantle-like sign in PLNTYs, and specific imaging patterns in BRAF-altered tumors, have emerging diagnostic utility. These imaging biomarkers offer non-invasive means to predict underlying molecular alterations, potentially guiding surgical planning and therapeutic decision-making. As pLGGs are predominantly "one-pathway diseases" with a single MAPK pathway alteration, targeted therapies directed at this pathway hold promise for improving outcomes, particularly in cases where complete resection is not feasible. The application of artificial intelligence further enhances our ability to detect subtle radiological patterns that correlate with specific genetic alterations. This radiogenomic approach not only advances diagnostic accuracy but also facilitates personalized treatment strategies, ultimately improving the management and prognosis for children with these challenging tumors. Continued research in this rapidly evolving field will undoubtedly refine our understanding of genotype-phenotype correlations and lead to more effective, molecularly targeted therapeutic interventions for pediatric glioma patients.
